# A Retrospective Analysis on Two-week Short-course Pre-operative Radiotherapy in Elderly Patients with Resectable Locally Advanced Rectal Cancer

**DOI:** 10.1038/srep37866

**Published:** 2016-11-25

**Authors:** Chen Shi, Hao Zhou, Xiaofan Li, Yong Cai

**Affiliations:** 1 Key Laboratory of Carcinogenesis and Translational Research (Ministry of Education/Beijing), Department of Radiation Oncology, Peking University Cancer Hospital and Institute, Beijing, China; 2 Department of Cardiology, Chinese PLA General Hospital, Beijing, China

## Abstract

To validate that a two-week short-course pre-operative radiotherapy regimen is feasible, safe, and effective for the management of elderly patients with locally advanced rectal cancer (LARC), we retrospectively analyzed 99 radiotherapy-naive patients ≥70 years of age with LARC. Patients received pelvic radiation therapy (3D-CRT 30Gy/10f/2w) followed by TME surgery; some patients received adjuvant chemotherapy. The primary endpoint was OS, while the secondary endpoints were DFS, safety and response rate. The median follow-up time was 5.1 years. The 5-year OS and DFS rates were 58.3% and 51.2%, respectively. The completion rate of radiotherapy (RT) was 99.0% (98 of 99). Grade 3 acute adverse events, which resulted from RT, occurred in only 1 patient (1.0%). In addition, no grade 4 acute adverse events induced by RT were observed. All 99 patients (100%) were able to undergo R0 surgical resection, and 68.6% of the patients received sphincter-sparing surgery. The rate of occurrence of clinically relevant post-operative complications was 12.1%. Three patients (3.0%) achieved pathologic complete responses, and forty-three patients (43.4%) achieved pathologic partial responses. The rates of T-downsizing and N-downstaging were 30.3% and 55.7%, respectively. Therefore, we believe that a two-week short-course pre-operative radiotherapy is feasible in elderly patients with resectable LARC.

Colorectal carcinoma, which is one of the most prevalent cancers worldwide, is the second leading cause of cancer-related death in most developed countrie^1^. Rectal carcinoma accounts for approximately 60% of all colorectal malignancies in China, and it carries a poorer prognosis than colon cancer. The number of elderly patients with rectal cancer in China has also increased in recent years.

Since the late 1980s, surgery alone has been considered the cornerstone of curative treatment for patients with rectal cancer^2^. Total mesorectal excision (TME) was performed worldwide as the standard surgical procedure for rectal cancer^3^. Nevertheless, surgery alone cannot result in the best survival outcomes, especially due to the high risk of local recurrence, particularly in cases of locally advanced rectal cancer (LARC, Stage II or Stage III, according to the American Joint Committee on Cancer, AJCC). In the early 1990s, post-operative chemo-radiation or moderate dose pre-operative radiotherapy was shown to significantly reduce local recurrence and to prolong overall survival^4^. Neoadjuvant radiotherapy (RT) then became the new standard treatment for locally advanced operable rectal cancers^5^.

The neoadjuvant radiotherapy regimen for locally advanced rectal cancer includes pre-operative short-course radiotherapy (represented by five fractions of 5Gy over 1 week) and pre-operative long–course chemo-radiotherapy (CRT) consisting of 5-fluorouracil (5-Fu) or capecitabine. In recent years, the short-course pre-operative radiation regimen for LARC was tested extensively in randomized trials^6^,^7^,^8^,^9^. This treatment resulted in a relative reduction in local recurrence of approximately 60% compared with surgery alone and was associated with acceptable toxicity.

Few studies have directly compared pre-operative short-course radiation and pre-operative long-course chemo-radiotherapy. One randomized study of 312 patients in Poland found no differences in local recurrence or survival^7^. Similarly, an Australian/New Zealand trial (TROG trial 01.04) that randomized 326 patients to short-course radiation or long-course chemo-RT found no differences in local recurrence and OS rates. Long-term results of the TROG 01.04 trial were recently published. In addition to the lack of a significant difference between 3-year local recurrence rates and 5-year OS rates, the late toxicity rates, distant recurrence, and relapse-free survival were not significantly different between the arms^9^. Overall, it appears that short-course RT provides effective local control and the same OS as long-course CRT schedules, and therefore may be an appropriate choice in some situations.

However, the existing evidence described above was mainly based on studies on young people or studies that did not conduct age subgroup analyses. To our knowledge, even fewer studies have assessed the outcomes of elderly patients with rectal cancer who received neo-adjuvant CRT or RT. As we know, the risks of side-effects with chemotherapy drugs can often exacerbate the damage caused by radiotherapy when patients are treated with concurrent chemo-radiotherapy, which may lead to poor tolerance of radiotherapy, especially in patients older than 70 years and those with co-morbidities. Some evidence has shown that less acute toxicity was observed in patients, including elderly patients, after short-course radiation compared with long-course chemo-radiation^10^. Thus, it is reasonable to suppose that, for elderly patients, short-course radiation followed by TME surgery may be feasible.

Nevertheless, 5*5Gy short-course pre-operative radiotherapy has been reported to cause chronic lumbosacral plexopathy^11^. Therefore, to avoid the late toxicity caused by the high doses in a single fraction, the Chinese Anti-Cancer Association (CACA) designed an intermediate pre-operative RT regimen (30Gy/10f/2w, which has a biologically equivalent dose to that of the regimen of 5*5Gy). Some clinical trials conducted at our hospital showed that this regimen led to a favorable curative effect^12^,^13^. This retrospective analysis aims to validate our hypothesis that this two-week pre-operative radiotherapy regimen is feasible, safe, and effective for the management of resectable locally advanced rectal cancer in elderly patients (≥70 years old).

## Methods and Materials

All experimental protocol in this study involving human participants were approved by the Ethic Committee of Peking University Cancer Hospital (Beijing, China), with the following reference number: 33135468. The informed consent forms, including radiation consent, operation consent and chemotherapy consent, were obtained from all individual participants included in this study. The methods were carried out in accordance with the Declaration of Helsinki and the guidelines of the Ethical Committee of the Cancer Hospital (Beijing, China).

Patients ≥70 years of age who had previously untreated clinical stage II or stage III rectal cancer and a histopathologically confirmed diagnosis of adenocarcinoma of the rectum, located within 10 cm from the anal verge, were eligible. The disease was staged according to the American Joint Committee on Cancer (AJCC) staging system. Other eligibility criteria included a lack of severe hematologic abnormalities. Exclusion criteria were as follows: clinical stage at diagnosis other than stage II-III, relapse or synchronous malignancies, a history of prior pelvic RT, tumor distance from the anal verge greater than 10 cm, and contraindications for surgery. In total, 99 eligible patients with a median age of 75 years (range, 70 to 82) with a diagnosis with LARC were treated at our hospital between December 2007 and December 2009. We analyzed the data of these patients in this article.

### Preoperative diagnosis

Locally advanced rectal cancer without distant metastasis was confirmed by the following methods: barium enema, colonoscopy including histopathological evaluation, magnetic resonance imaging (MRI) or computed tomographic (CT) scans of the pelvis, and CT scans of the chest and abdomen. The proportion of patients with MRI scans was 51.5% (51 of 99 patients). Endo-rectal ultrasonography was optional. Lymph node metastasis was defined as the shortest lymph node diameter of 5 mm or greater on any imaging technique. Tumor location was classified according to the distance from the anal verge (either >5 cm or ≤5 cm).

### Radiation therapy

Radiation therapy, which consisted of external irradiation, was administered with the three-field 3D-CRT technique in fractions of 3.0Gy/day, 5 days per week for 2 weeks. The total dose of radiation was 30Gy (30Gy/10f/2w). The radiation fields included 1 posterior field and 2 lateral fields. The clinical target volume in this study included the primary disease, regional lymphatic areas (perirectal lymph nodes and internal iliac nodes), the mesorectum, presacral and perineal regions, and for distant tumors, the anal sphincter. The external iliac nodes were electively irradiated when the bladder, prostate or some gynecologic organ demonstrated clinical invasion or when the external iliac node was clinically positive. Multiple convergent fields, beam conformation with blocks, immobilization devices, high-energy photons from linear accelerators, and conventional simulation with CT scan with dosimetric evaluation were used in treatment planning for all patients.

### Surgery

Surgical resection according to the principles of TME was performed two weeks after the patients completed pre-operative treatment. The completeness of resection was scored as R0 for negative margins, R1 for microscopic involvement of margins, and R2 for gross residual tumor.

### Adjuvant chemotherapy

Post-operative adjuvant chemotherapy was recommended if it could be tolerated by the patients. It was recommended for patients with ypN+ lymph node metastasis. The regimen used was 6 months of capecitabine, 10–12 cycles of FOLFOX6 or 6–8 cycles of XELOX.

### Toxicity

During the RT treatment, the hematologic, urinary, anorectal and dermatologic toxicities were tested every 7 days by blood, urine, and dermatologic assessments. Toxic effects were evaluated according to the National Cancer Institute Common Terminology Criteria for Adverse Events (CTCAE), version 3. If uncontrollable grade 3 toxicity was detected (with supportive treatment), radiation was suspended until adverse events could be reversed to grade 2. If grade 3 toxicity persisted for more than 1 week, despite interruption of treatment and supportive therapy, radiation was terminated and surgery was scheduled.

### Evaluation of pathological specimens

After surgery, the responses of tumors to pre-operative radiotherapy including pathological stage and residual tumor volume were evaluated by histopathological examination. The responses were evaluated based on the system used to grade tumor response established by Ryan R, *et al*. with modifications. Briefly, complete, moderate, minimal, and poor response corresponded to grades of 0, 1, 2 and 3, respectively. Pathologic complete response (pCR) was defined as a complete absence of tumor cells from the resected specimen (ypT0) and the resected nodes (ypN0). Other indicators included T-downsizing and N-downstaging. T-downsizing was defined as a reduction in relation to the pre-treatment clinical T stage, while N-downstaging was defined as a reduction in relation to the pretreatment clinical N stage.

### Follow-up

All of the patients were reviewed at routine follow-up, which was usually 6 weeks after surgery and every 3 months during the initial 2 years. During years 3 to 5, patients were assessed every 6 months, and after 5 years, they were assessed every 12 months. In addition, patients were instructed to return if they experienced unusual symptoms. The baseline assessment included patient’s medical history and physical examinations, tumor measurements, assessment of performance status, measurement of carcinoembryonic antigen (CEA) levels, hematologic studies, and serum chemical analysis; CT or MRI of the pelvis, chest radiography or CT, and abdominal ultrasonography or CT were also performed. When signs or symptoms of possible recurrent disease were present, relevant radiologic tests including barium enema and endoscopic images (e.g., colonoscopic examination) were requested. The diagnosis of recurrence was based on the results of imaging studies and, if possible, cytological analysis or biopsy results. Local recurrence was defined as a progressive change on CT or MRI in addition to pelvic symptoms, a rise in the CEA level with confirmed activity on PET-CT, or as a biopsy-proven pelvic recurrence within the pelvic irradiation field.

### Endpoints and statistical analyses

Statistical analyses of all collected data were performed using SPSS (version 17.0) statistical software. The primary end point of the analysis was overall survival (OS). Secondary end points were the safety (incidence of toxic effects and complications after surgery), the response rate (pCR, T- and N-downstaging), the rate of sphincter preservation, the local recurrence rate and disease-free survival (DFS). As of June 2015, surviving patients had been followed for a median of 5.1 years (range, 13 months to 6.5 years). OS was defined as the time from enrollment to the time of death from any cause. DFS was defined as the time from enrollment to the diagnosis of recurrence or metastasis. The Kaplan-Meier survival curve was used to estimate the proportion of patients who survived or remained disease free at each interval.

## Results

Table 1 shows the clinical characteristics of the 99 patients ≥70 years old with clinical stage II/III rectal cancer.

### Primary endpoint

**Figure 1:**
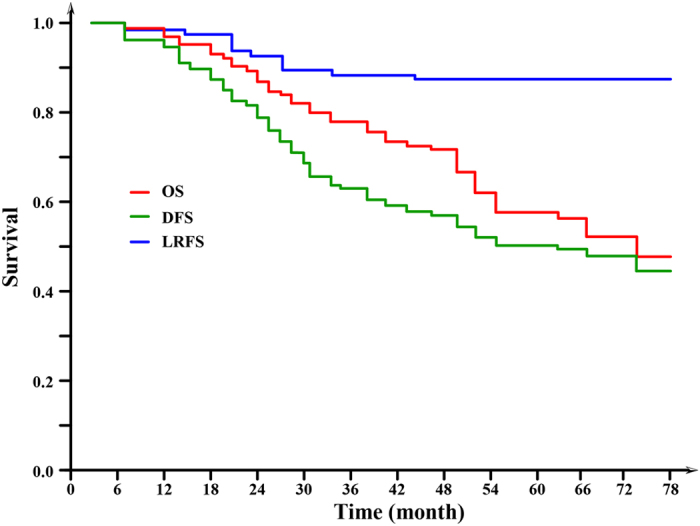
Kaplan-Meier Curve of Local Recurrence-free Survival (LRFS), Disease-free Survival (DFS), and Overall Survival (OS). The 5-Year LRFS, DFS, and OS Was 87.9%, 58.3%, and 51.2%, Respectively.

All of the 99 patients had received follow-up. At the time of this analysis, 41 of the patients died during follow-up; 20 deaths were related to rectal cancer, 8 were related to other cancers, 12 were related to other causes and 1 was due to an unknown cause. The 5-year overall survival (OS) was 58.3% (Fig. 1).

### Local recurrence rate and DFS

During follow-up, 11 patients (11.1%) had a local recurrence, 4 patients were stage II and 7 patients were stage III. The 5-year local recurrence-free survival (LRFC) was 87.9% (Fig. 1). And 37 patients (37.4%) had distant metastases, 9 patients were stage II and 28 patients were stage III. The metastases occurred in the lung (n = 9), liver (n = 8), peritoneum (n = 4), cerebrum (n = 3), bone (n = 3), lung and liver (n = 3), lung and cerebrum (n = 3), and liver and cerebrum (n = 2), while multiple organ metastases also occurred (n = 2). The 5-year disease-free survival (DFS) was 51.2% (Fig. 1).

### Toxic effects of RT

Of the 99 patients, 98 (99.0%) completed the two-week short-course pre-operative radiotherapy regimen. RT toxicity data were collected, which showed that 34 patients (34.3%) experienced one or more than one type of acute adverse event, and only 1 patient had a grade 3 adverse event. No grade 4 toxicity was observed. The total frequency of acute adverse events due to RT was 42, and these included fatigue (n = 4), diarrhea (n = 19), hematologic events (n = 6; including leukopenia, neutropenia, thrombocytopenia), dermatologic (n = 12), and genitourinary events (n = 1). No deaths were associated with RT. Only 1 patient experienced an interruption in radiation therapy due to incomplete intestinal obstruction (21Gy/7f). Fortunately, this patient underwent successful radical surgery and satisfactory post-operative rehabilitation. The acute RT toxicity data of the patients are listed in Table 2.

A univariate analysis was performed for the 19 patients who experienced the RT-induced acute adverse effect of diarrhea. The influencing factor for diarrhea may have been the distance from the anal verge, but the distances were not significantly different between those with and without diarrhea. (Table 3).

A univariate analysis was also conducted in the 12 patients who experienced the RT-induced acute dermatologic adverse effects. The significant influencing factor for dermatologic events was diabetes. (Table 4).

### Surgery and post-operative complications

The average interval between radiation and surgery was 2 weeks (range, 10–38 days; median, 17 days). No deaths occurred prior to surgery. All of the 99 patients underwent the R0 TME surgical resection. In the 65 patients with low rectal cancer (distance from the anal verge ≤5 cm), 25 patients underwent abdominoperineal resections (APR), 38 patients underwent low anterior resections (LAR), and 2 patients underwent Hartmann’s procedure. The rate of anal sphincter preservation in low rectal cancer was 58.5%. In the 34 patients with rectal cancer with a distance from the anal verge >5 cm, 4 patients underwent an APR and 30 patients underwent an LAR. In total, 68 patients received sphincter-sparing surgery, and overall the rate of sphincter preservation was 68.6%. In all, 12.1% of patients experienced clinically relevant post-operative complications, the most frequent of which were anastomotic leakage (5.1%), abscess of the pelvic cavity and obstruction of the ileum (2.2%) (Table 5).

A univariate analysis was performed in the 5 patients with anastomotic leakage. The significant influencing factors for the development of this complication were diabetes and the degree of the American Society of Anesthesiologists (ASA) (Table 6).

### Pathologic response rate to pre-operative radiotherapy

The pathologic response was assessable in all patients. Of the 99 patients, 3 (3.0%) achieved a pathologic response of ypCR and were considered to be free of disease, while 43 (43.4%) achieved a pathologic response of ypPR. When the primary tumor response was evaluated according to the method of Ryan *et al*., 3 of 99 patients (3.0%) achieved a complete pathologic response (grade 0), and 43 (43.4%) still had minute residual tumor cells (grade 1). The rate of T-downsizing was 30.3% (30 of 99 patients, 11 of 20 in stage II and 19 of 79 in stage III). Nodal status was evaluated in all 99 patients, and the rate of N-downstaging was 55.7% (44 of 79 N+ patients).

## Discussion

Although surgery remains the primary treatment for rectal cancer, the current consensus dictates that pre-operative treatments help to achieve optimal outcomes^14^. The reported advantages of neo-adjuvant treatment are the reduction in local recurrence rates^15^, the ability to perform surgery in patients with stage II or III rectal cancer and an increase in OS and DFS.

Nevertheless, most clinical studies have not emphasized elderly patients, and thus research on elderly patients with rectal cancer has not really been developed worldwide. Patients older than 65 years have often been underrepresented, and those older than 70 or 75 years have been altogether excluded from randomized trials, which limits the ability to evaluate the risk/benefit ratio of neo-adjuvant RT or CRT for the elderly. Some nonrandomized data have suggested that neo-adjuvant therapy was associated with a survival advantage in patients with stage III rectal cancer who are older than 65 years^16^,^17^. However, further efforts are still needed.

As we know, elderly patients are a special category of people, who often have hypo-immunity, higher comorbidity burden and lower functional status of vital organs such as the heart, liver, and kidney to name a few. Concurrent chemo-radiotherapy may increase the treatment-related toxicity. Therefore, this treatment should be carefully considered in older patients or patients with co-morbidities. Moreover, in China, because of financial constraints, compared with long-course RT, short-course RT may be better accepted by older individuals. Moreover, chemotherapy was associated with lower “social functioning” and lower scores on the health-related Quality of Life Scale^18^. For these reasons, we believe that neoadjuvant RT may be more appropriate for elderly patients with LARC.

This two-week short-course neoadjuvant radiotherapy regimen was considered sufficiently safe in elderly patients, and it was associated with a high rate (99.0%) of completion. No grade 4 acute adverse events were observed. Moreover, the incidence of grade 3 adverse events was only 1%, and the rate of post-operative complications was 12.1%. These rates are relatively low, and most of these events could be easily managed by conservative therapy. Based on previous findings, acute toxicity during short-course radiotherapy or 3–7 days after the completion of radiotherapy, most often of grade 1–2 severity, occurred in approximately 10–41% of patients. Gastrointestinal symptoms or perianal pain were observed most frequently^10^,^19^,^20^,^21^,^22^,^23^. Our results were consistent with these data. The rate of severe acute toxicity (grade 3–4) was reported in approximately 2–13% of patients^10^,^19^,^20^,^21^,^22^,^23^. The occurrence of post-operative complications was approximately 15–45% of patients^24^. Our results were comparatively lower than these data.

A univariate analysis showed that diabetes or the degree of ASA was a significant influencing factor for the development of acute RT-related adverse events or post-operative complications; this hints that the tolerance to the regimen in this study may be primarily related to the comorbidity burden or to the functional status of the patients. Therefore, we believe that the analyses and medical interventions during the perioperative period are necessary as part of an important strategy to reduce toxic side effects and complications.

Therefore, from the perspective of the safety of this regimen, this neoadjuvant RT regimen is well tolerated by elderly patients, which is consistent with what we initially hypothesized.

Actually, the feasibility of clinical treatment regimen not only depends on its safety, but it also depends on a good curative effect, which is even more important. At the median follow-up period of 5.1 years, the OS rate was 58.3%, and the DFS rate was 51.2%. Compared with previous studies, these results could be considered to represent an instance of good survival outcomes of patients with LARC who received neo-adjuvant RT. The EORTC (European Organization for Research and Treatment of Cancer) 22921 trial reported that in the groups that received pre-operative radiotherapy, the 5-year overall survival was 64.8%, and the 5-year disease-free survival was 54.4%^25^.

In addition, the rates of sphincter preservation, T-downsizing, and N-downstaging in our study were also satisfactory. In a retrospective trial that evaluated the outcome of patients with rectal cancer who were unfit for chemotherapy but who received classic short-course preoperative radiotherapy with TME surgery, the rate of sphincter preservation was 57%^19^. Another phase II trial of 5*5Gy short-course radiotherapy followed by TME surgery for loco-regionally advanced rectal cancer reported a sphincter preservation rate of 73%. However, the patients are still being followed-up, and data on survival outcome are not yet available^26^.

Honestly, the unfavorable outcome of this analysis was the pCR. Some evidence has shown that pCR after short-course radiotherapy was most often observed in approximately 10% of patients and varied between 1 and 12%^27^. In our analysis, the pCR rate was 3.0%. This may be due to the shorter interval between radiation and surgery. The incomplete pronounced effects of the radiation may not shrink the tumors completely. Within a few days following the start of radiation, non-viable cancer cells appear morphologically intact, and no down-staging occurs. If the interval until surgery is extended, non-viable cancer cells undergo lysis. However, the extension of the rest period between radiation and surgery does not cause additional DNA damage because it occurs only at the time of irradiation. This may explain why a lack of down-staging after short-course radiotherapy and immediate surgery does not confer a poor prognosis.

On the contrary, some evidence has shown that a higher rate of pCR was observed in the chemo-radiation group compared with the short-course group, but no differences were found in the DFS or OS rates. A pooled analysis of the EORTC 22921 trial data and the FFCD (Federation Francophone de Cancerologie Digestive) 9203 trial data compared the pre-operative fluorouracil-based CRT (n = 886) with radiotherapy alone (n = 881). This comparison showed that the ability of patients to attain a pCR was significantly improved by pre-operative CRT; however, the effects did not translate into improved progression-free or overall survival^2^. The 10-year long-term results of the EROTC 22921 trial also revealed no differences in the DFS and OS between the pre-operative RT and CRT groups^28^. Moreover, the impact of a radical R0 resection may have a relevant influence on the prognosis. Some trials have indicated that surgical margins seem to be more important to the end point^29^,^30^. In summary, we agree that pCR is not a reliable end point in the comparison of the efficacy of different treatment regimens on long-term outcomes.

As mentioned, the two-week short-course pre-operative RT regimen let to effective outcomes. In Wang L’s study, the 5-year DFS and OS were 79.0% and 73.5%, respectively, which higher than our analysis and other researches^13^. However, the elderly patients (≥70 years old) in Wang L’s study only made up the minority (about 10%), while the patients in our study were all elderly. And therefore, the patients in our study are largely not the same as Wang’s study. As we know, the elderly patients of rectal cancer have worse prognosis. Thus, we suppose the better survival outcomes of Wang L’s study may attribute to younger patients eligible. Then we are afraid Wang L’s study couldn’t truly reflect the risks/benefits of this neo-adjuvant treatment regimen in elderly patients. But that is just what our study showed.

Briefly, this two-week short-course pre-operative RT regimen used in our study led to effective outcomes and good tolerance. All of the patients eligible were ≥70 years old. In our opinion, we provide a feasible option for neoadjuvant treatment for elderly patients (≥70 years old) with resectable locally advanced rectal cancer. Our data contribute to the limited evidence about the risks/benefits of neo-adjuvant treatment in elderly patients with LARC. Additionally, this treatment is cost-effective and convenient, and could effectively improve patient compliance to the regimen.

Of course, the limitations of this study must also be acknowledged. First, this retrospective analysis did not include a randomized control group for a comparison of the results. Second, as the study tested a method of patient selection, the patients selected were only from one hospital, and thus there may have been an inherent selection bias. Third, considering the higher influence of RT-related side effects and post-operative complications on the life quality of these elderly patients, we should have quantified the quality of life of these patients via the incorporation of a rating scale into our study design. In a subsequent study, we will conduct follow-up visits for these patients to determine their late RT-related toxicities and long-term survival outcomes. Additionally, we are carrying out a randomized controlled clinical trial to compare neo-adjuvant RT and CRT in elderly patients with locally advanced rectal cancer.

## Conclusions

In elderly patients with rectal cancer, the two-weeks short-course pre-operative radiotherapy regimen followed by TME surgery led to improved survival outcomes in terms of DFS and OS. Furthermore, the few adverse effects and post-operative complications were tolerable in these patients. Overall, this regimen is considered a feasible treatment for resectable locally advanced rectal cancer in elderly patients.

## Additional Information

**How to cite this article**: Shi, C. *et al*. A Retrospective Analysis on Two-week Short-course Pre-operative Radiotherapy in Elderly Patients with Resectable Locally Advanced Rectal Cancer. *Sci. Rep*. **6**, 37866; doi: 10.1038/srep37866 (2016).

**Publisher's note:** Springer Nature remains neutral with regard to jurisdictional claims in published maps and institutional affiliations.

## Figures and Tables

**Table 1:** Clinical characteristic of 99 elderly patients with rectal cancer.

Clinical characteristic	Number of patients	Percentage of patients
Sex		
Male	56	56.6%
Female	43	43.3%
Number of comorbidities		
0	62	62.6%
1	25	25.3%
≥2	12	12.1%
Clinical stage T		
T2	15	15.1%
T3	66	66.7%
T4	18	18.2%
Clinical stage N		
N0	20	20.2%
N1	23	23.2%
N2	56	56.6%
Distance from anal verge		
≤5 cm	65	65.7%
>5 cm	34	34.3%

**Table 2:** The frequencies of acute RT toxicities.

Toxicity	I°	II°	III°	IV°	Total
Fatigue	4	0	0	0	4
Hematologic	5	1	0	0	6
Diarrhea	14	4	1	0	19
Dermatologic	8	4	0	0	12
Genitourinary	1	0	0	0	1
Total	32	9	1	0	42

**Table 3:** Univariate analysis of RT diarrhea.

Characteristic	Number of patients	Number of diarrhea	X2 value	P value
Sex				
Male	55	13	1.448	0.229
Female	43	6		
Comorbidities				
0	62	12	0.089	0.957
1	24	5		
≥2	12	2		
Diabetes				
No	79	17	0.585	0.444
Yes	19	2		
Clinical stage T				
T2	14	5	2.786	0.248
T3	66	11		
T4	18	3		
Clinical stage N				
N0	20	2	2.722	0.256
N1	22	3		
N2	56	14		
From anal verge				
≤5 cm	65	9	3.793	0.052
>5 cm	33	10		

**Table 4:** Univariate analysis of RT dermatologic.

Characteristic	Number of patients	Number of diarrhea	X2 value	P value
Sex				
Male	55	7	0.027	0.869
Female	43	5		
Diabetes				
No	79	5	10.583	0.001*
Yes	19	7		
Clinical stage T				
T2	14	3	1.849	0.397
T3	66	8		
T4	18	1		
Clinical stage N				
N0	20	2	0.938	0.626
N1	22	4		
N2	56	6		
From anal verge				
≤5 cm	65	7	0.090	0.765
>5 cm	33	5		

^*^Fisher’s exact text.

**Table 5:** Surgery tolerance and complications management.

Clinical characteristic	Number of patients	Percentage of patients
Surgical complications		
No	87	87.9%
Yes	12	12.1%
Type of complications		
Anastomotic leakage	5	5.1%
Abscess	2	2.0%
Obstruction/Ileum	2	2.0%
Infection	1	1.0%
Fistula	1	1.0%
Other	1	1.0%
Treatment		
Conservative	9	75.0%
Surgical	3	25.0%

**Table 6:** Univariate analysis of anastomotic leakage.

Characteristic	Number of patients	Number of anastomotic leakage	X2 value	P value
Sex				
Male	56	3	0.092	0.761
Female	43	2		
Diabetes				
No	80	2		0.047*
Yes	19	3		
Clinical stage T				
T2	15	1	0.126	0.939
T3	66	3		
T4	18	1		
Degree of the ASA				
I-II	81	2		0.041*
III-IV	18	3		

^*^Fisher’s exact text.
